# The impact of limited healthcare access among patients with light chain and transthyretin amyloidosis: real-world survey during COVID-19 lockdown period in France

**DOI:** 10.1186/s13023-025-03859-1

**Published:** 2025-07-08

**Authors:** D. Guijarro, A. Jobbe-Duval, S. Aguilhon, F. Bauer, E. Donal, J. C. Eicher, J. Jeanneteau, B. Gellen, D. Kenizou, O. Lairez, B. Lequeux, D. Legallois, P. Réant, M. Salvat, M. F. Seronde, M. Kharoubi, A. Whereat, A. Farrugia, C. Taieb, A. Zaroui, T. Damy

**Affiliations:** 1 Cardiovascular Institute, Groupe Hospitalier Mutualiste de Grenoble, Grenoble, France; 2 Heart Failure Service, Assistance and Transplantation, Hospital Louis Pradel, Bron, France; 3 Cardiology, University Hospital Montpellier, Montpellier, France; 4 Cardiology, University Hospital Charles Nicolle, Rouen University Normandy, Rouen, France; 5 Cardiology, University Hospital Rennes, Pontchaillou Hospital, Rennes, France; 6 Cardiology, University Hospital Dijon, Dijon, France; 7 Cardiology, Clinique Saint Joseph, Trélazé, France; 8 Cardiology, Polyclinique de Poitiers, Poitiers, France; 9 Cardiology, Hospital Emile Muller, Mulhouse, France; 10 Cardiology, University Hospital,Toulouse Rangueil Hospital, Toulouse, France; 11 Centre Cardio-Vasculaire, CHU de Poitiers, Site de La Milétrie, Poitiers, France; 12 Department of Cardiology, Normandy University, UNICAEN, INSERM U1086 ANTICIPE, CHU de Caen-Normandy, Caen, France; 13 Cardiology, University Hospital de Bordeaux, Bordeaux, France; 14 Cardiology, University Hospital Grenoble, Grenoble, France; 15 Cardiology, University Hospital Besançon, Besançon, France; 16 Cardiology Department and French National Reference Centre for Cardiac Amyloidosis, GRC Amyloid Research Institute and Clinical Investigation Centre 1430 at University Hospital Henri-Mondor AP-HP, and IMRB, INSERM, University Paris Est Creteil, 94010 Créteil, France; 17 Speak the Speech Consulting, Asnières-sur-Seine, France; 18 Association Française Contre l’Amylose, Marseille, France; 19 Emma Clinic, Fontenay-sous-Bois, France

**Keywords:** Burden, Cardiac amyloidosis, Care pathway, COVID-19, Self- reported questionnaire, Patient experience

## Abstract

**Background:**

The containment strategies during the COVID-19 pandemic between December 2019 and 2022 significantly disrupted the healthcare system. Cardiac amyloidosis has a poor prognosis and requires frequent follow-up in reference centres.

**Objectives:**

To assess the impact of limited access to healthcare on the patient burden and care pathway in France.

**Method:**

This cross-sectional, self-questionnaire survey was conducted between June and October 2021 among cardiac amyloidosis patients registered at Expert Centres of the French Amyloidosis Network.

**Results:**

Overall, 1015 patients participated of whom, 229 had light chain amyloidosis, 786 had transthyretin amyloidosis. Disrupted clinical follow-up was reported in 21.1% of respondents, 15% had follow-up visits postponed. No alternative follow-up option was proposed for 45% of these patients. Few patients reported treatment discontinuation (Light chain (1.1%), transthyretin (1.3%). Significantly more newly diagnosed light chain (37.9%) than transthyretin amyloidosis patients (30.4%) reported the containment strategies caused a poor initial work-up experience (*p* = 0.034). Among those patients who reported a COVID19 infection (9.7%) more patients with light chain amyloidosis (75.0%) were hospitalized than transthyretin amyloidosis (37.1%), (*p* = 0.006). Only 587 (57.0%) patients answered vaccination question, most (92.0%) reported having been vaccinated.

**Conclusion:**

Patients with light chain amyloidosis reported having had a higher impact to their care management than transthyretin amyloidosis patients during the COVID19 pandemic containment periods.

**Supplementary Information:**

The online version contains supplementary material available at 10.1186/s13023-025-03859-1.

## Introduction

**Fig. 1:**
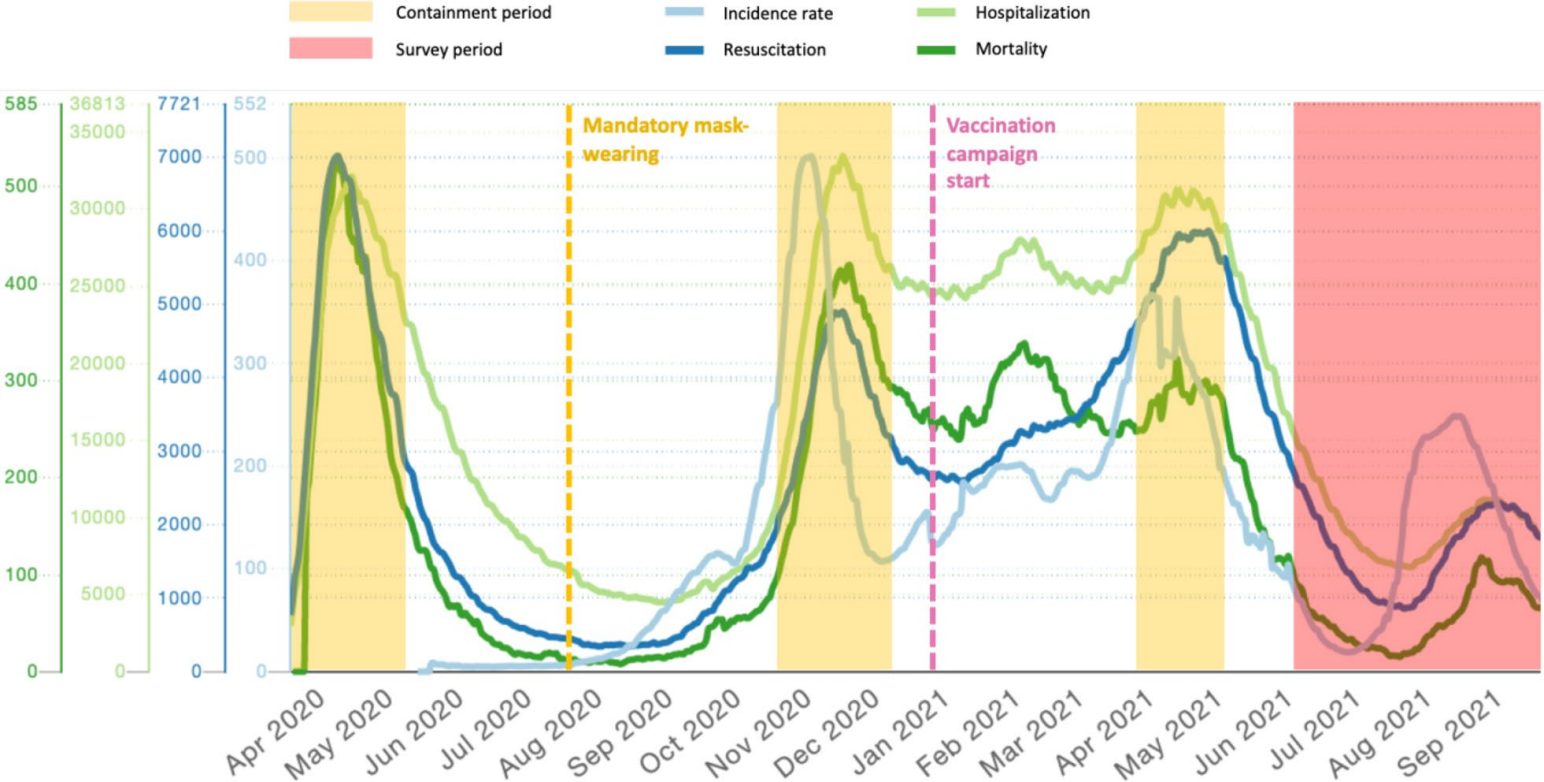
Negative impact of COVID-19 restrictions. Figure 1 illustrates the zero COVID-19 strategy and lockdown periods. French public health data from the National institute of statistical and economic research (INSEE) illustrates incidence during the various COVID-19 lockdown periods in France. The survey started shortly after the last lockdown in France. Incidence rate: number of cases per week per 100,000 inhabitants; Resuscitation: number of intensive care beds occupied; Hospitalization: number of hospital beds occupied for COVID-19; Mortality: daily number of hospital deaths linked to COVID19. https://covidtracker.fr/

The COVID-19 pandemic resulted in substantial social and organizational changes from December 2019 to December 2022 worldwide. In France, a zero COVID-19 strategy was initially implemented, involving successive lockdowns, curfew periods and mask wearing until the national vaccination program began (Fig. 1). The consequences of these measures in the French population reduced physical activity [1], modified eating habits and particularly mental health within the French population in general [2].

The zero COVID-19 strategy severely disrupted medical health care services [3] with COVID-19 testing and organizational modifications that caused preplanned hospital or community consultations to be cancelled or in some cases replaced with teleconsultation. The purpose of cancelling healthcare consultations was to limit the contact between patients, health and administrative staff to avoid spreading SARS-CoV-2 infection. [4–7].

Cardiac amyloidosis (CA) is a severe disease caused by amyloid light chain (AL-CA) or transthyretin (ATTR-CA) fibrils that infiltrate cardiac tissue [8]. Patients with CA usually experience heart failure symptoms, reduced quality of life and have a poor survival prognosis [9]. AL-CA is typically a more severe form of the disease that requires complex diagnostic examinations and treatment. The main treatment is chemotherapy, which aggravates immune suppression increasing the risk of infection and requiring more frequent follow-up than ATTR-CA patients [10]. Concerning ATTR-CA, the non-invasive diagnostic strategy, validated since 2016 [11], has greatly facilitated diagnosis but requires multimodal cardiac imaging. Moreover, when amyloidosis typing is necessary, cardiac or extra-cardiac biopsies and expertise in reference centers are often required. In France, access to the specific treatment with tafamidis has been available for patients since 2018, shortly after the results of the ATTR-ACT trial were presented [12]. Subsequently, the decree allowing tafamidis prescription at 61 mg was issued in May 2021, when prescribed by a hospital cardiologist. Therefore, access to a reference center is crucial for both diagnosis and treatment for patients, regardless of their type of cardiac amyloidosis. 

Considering that CA patients are vulnerable, the limited access to medical care may have further impacted the burden of diagnosis or management of their disease. Having an understanding about how AL-CA and ATTR-CA patients felt their quality of life was impacted during this time may influence the management of this chronic type of heart failure post pandemic.

The objective of this study is to explore the impact of the COVID-19 period on healthcare management among patients with AL-CA and ATTR-CA in France using a self-administered questionnaire.

## Materials and methods

### Study design, setting and population

This prospective, cross-sectional survey was conducted among current or newly diagnosed patients with any form of cardiac amyloidosis registered at any of the participating CA centres of reference. The study was conducted between 8th of June and 29th October 2021, after the initial COVID-19 lockdown period and the two subsequent containment periods, barrier implementations and vaccination schedules (Fig. 1).

### Data collection

Data were collected from a self-administered, study-specific questionnaire. The questionnaire was developed by a multidisciplinary, expert committee consisting of cardiologists specialized in managing CA, a patient representative and a methodologist (Supplementary Table 1). Our research group previously conducted a similar survey that explored other aspects of cardiac amyloidosis [9]. The experience gained from this previous work allowed for a smoother process, enhancing the reliability and consistency of the current study. The questionnaire was composed of 38 questions, divided into five sections: 4 questions concerning background, demographic and CA diagnosis, 5 questions concerning patients’ feelings when diagnosed with CA during the COVID-19 lockdown and subsequent containment periods, 17 questions regarding therapeutic management, follow-up and vaccination, 5 questions concerning patients infected with the SARS-CoV-2 virus and 7 questions concerning their relatives that could be infected by the SARS-CoV-2 virus.

Responses to questions were varied. The questionnaire contained some closed questions that allowed patients to describe their experience during the COVID-19 pandemic (participants were asked to choose one of several possible options, others were binary “yes” or “no” answers). Other questions used analog scales and Likert scales to rate the feelings and emotions they experienced in these situations (Supplementary Table 1).

Expert centres in France in collaboration with the French Amyloidosis Patient Association (‘Association Française Contre l’Amylose’ (AFCA)), the hereditary or rare cardiac disease (CARDIOGEN) network and the French Heart Failure and Cardiomyopathies group disseminated the anonymous questionnaire to patients with cardiac amyloidosis registered at any of the participating expert centres. Amyloidosis patients registered in the AFCA and referral centres databases were solicited to participate in the study. Registered or new patients who presented at participating centres were also proposed the study. The questionnaire was made available in cardiologist consulting rooms, online via a restricted website address or via direct emailing. Each center had also the option to duplicate it to ensure the widest possible dissemination. Therefore, the exact number of questionnaires distributed is unknown. Furthermore, there was no specific process to include patients based on the type of their amyloidosis. To determine the type of disease, a specific question was included in the questionnaire, and the type of amyloidosis was thus based on the patient's knowledge of their diagnosis. Ethics committee approval was not obtained considering the urgency of the situation and that the anonymous surveys about patient opinions that do not involve any medical procedures are tolerated under French law. The study was reported in accordance with the STROBE guidelines.

### Statistics

The responses from patients who completed the survey were classified according to the two most commonly occurring amyloidosis subtypes AL-CA and ATTR-CA. Responses to survey questions were calculated as a percentage of the number of answers.

Descriptive characteristics for the population were represented with quantitative continuous variables and presented as mean and standard deviation when normally distributed. Non-normally distributed data were described using medians (1st and 3rd quartile). Categorical variables were presented as numbers (%) The different aspects of healthcare impacted by COVID-19 were described for the whole population and comparisons between AL-CA and ATTR-CA populations will be performed using IBM® SPSS® statistic (Version 21), USA. The significance threshold for p-values was set at <0.05. Also, the impact will be illustrated in spider graphs in which each axe represents those dimensions that illustrate the negative impact of a COVID19-specific dimension that differed between amyloidosis subtypes.

Although participants were encouraged to complete the whole questionnaire, they were not obliged to answer all questions. Missing response data were disregarded when evaluating findings.

## Results

### Patient and disease characteristics

**Fig. 2:**
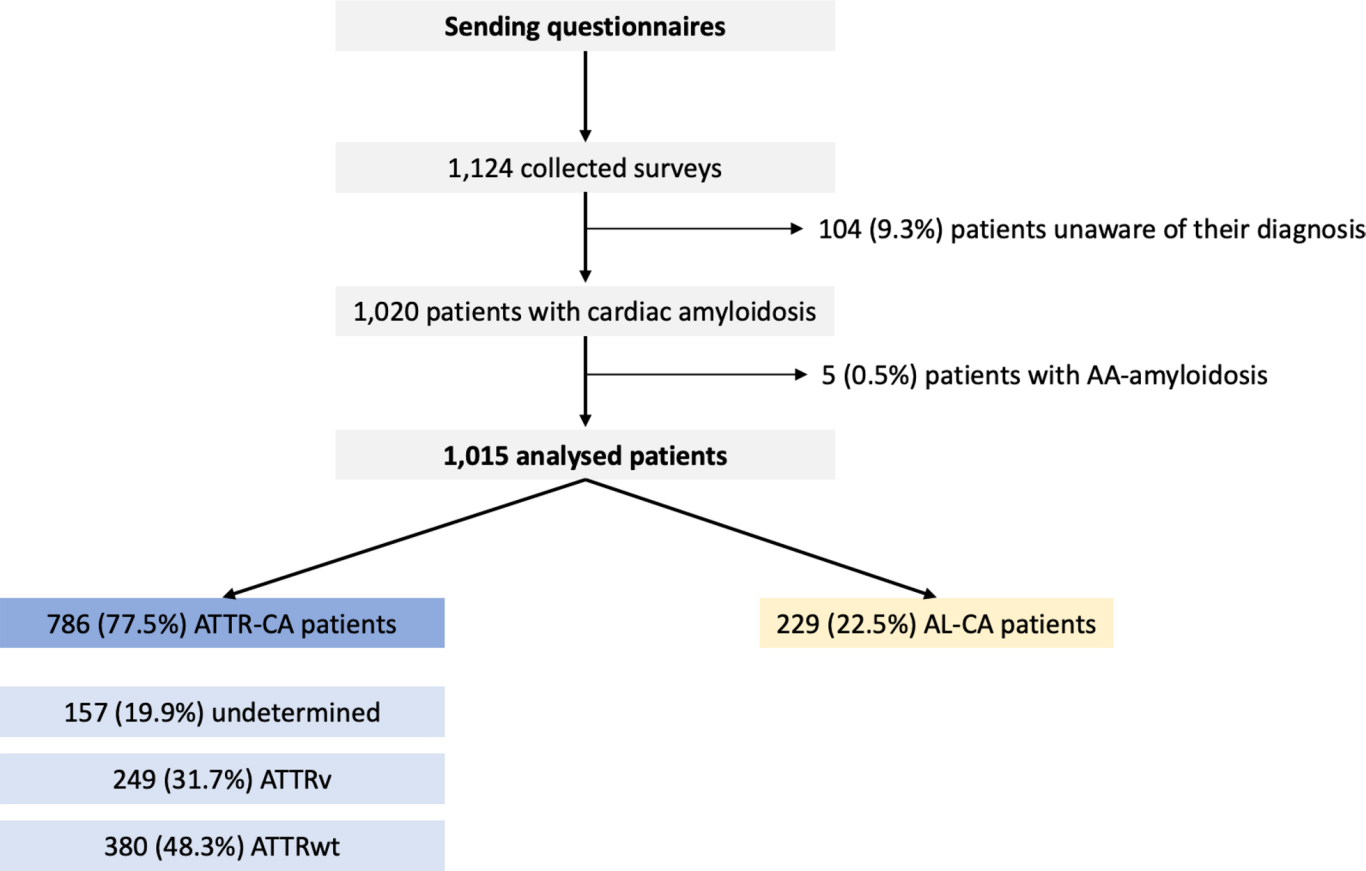
Flow Chart. The STROBE flow chart illustrates patient selection for analysis

A total of 1124 CA patients returned a questionnaire. Most were male with a median age of 71 years and 401 (40.6%) live in the Paris metropolitan area. Among these respondents, 5 had amyloid serum A amyloidosis and 104 were uncertain of their diagnosis and were excluded. The remaining 1015 questionnaires were analysed. Among these, 229 (22.6%) had confirmed AL-CA, 786 (77.4%) patients had ATTR-CA, of which around a third were hereditary (ATTRv) and nearly a half were wild type (ATTRwt) (Fig. 2).

Overall, among patients surveyed, around a fifth reported feeling that the health crisis caused by the COVID-19 pandemic significantly or very significantly disrupted their healthcare. Specifically, significantly more patients with AL amyloidosis felt their healthcare had been disrupted than those patients with ATTR amyloidosis (*p* = 0.0325, Table 1).

**Table 1:** The impact of the COVID-19 pandemic on patients with cardiac amyloidosis

Parameters and questions	Number of answers	All (n = 1015)	AL-CA (n = 229)	ATTR-CA (n = 786)	*p*-value
Age, years (mean, STD)	1001	71.85 (74)	65.5 (10.4)	73.7 (13.2)	** <.0001**
Male sex (%)	1110	752 (67%)	140 (61.7%)	612 (78.16%)	** <.0001**
*Area* of residence (% Paris metropolitan area)	989	401 (40.55%)	88 (41.12%)	313 (40.39%)	0.8464
*Diagnosis made during the health crisis*	1009	222 (22.00%)	52 (22.7%)	170 (21.8%)	0.7695
Bad experience					
Visit restrictions in case of hospitalization	123	58 (47.15%)	28 (60.9%)	30 (39.0%)	**0.0185**
Absence of a relative at the time of the announcement	115	45 (39.13%)	22 (55.0%)	23 (30.7%)	**0.0109**
Visit restrictions	117	62 (52.99%)	25 (62.5%)	37 (48.1%)	0.1375
Longer waiting times for medical appointments	91	37 (40.66%)	12 (63.2%)	25 (34.7%)	**0.0248**
Telehealth (visio or telephone)	54	14 (25.93%)	11 (47.83%)	3 (9.7%)	**0.0016**
*Impact on regular follow-up and treatment*					
Consultation postponed due to COVID-19	846	112 (13.24%)	29 (16.29%)	83 (12.43%)	0.1762
No alternatives suggested by your doctor	109	51 (46.79%)	10 (34.48%)	41 (51.25%)	0.1211
Consultation at EXPERT CENTER cancelled or postponed	788	140 (17.77%)	24 (13.41%)	116 (19.05%)	0.0827
No alternatives suggested by the EXPERT CENTER	129	56 (43.41%)	11 (47.83%)	45 (42.45%)	0.6374
Consultation at HOSPITAL cancelled or postponed	915	160 (17.49%)	36 (16.59%)	124 (17.77%)	0.6906
No alternatives suggested by the HOSPITAL	148	63 (42.57°%)	12 (35.29%)	51 (44.74%)	0.3284
Medical exams postponed due to COVID-19	911	110 (12.07%)	21 (9.81%)	89 (12.77%)	0.2457
Hospitalization to monitor the disease cancelled or postponed	497	67 (13.48%)	19 (14.39%)	48 (13.15%)	0.7200
Do you have oral or injectable treatment for your amyloidosis?	1003	835 (83.25%)	152 (66.67%)	683 (88.13%)	** <.0001**
Have you stopped taking your amyloidosis medication?	834	9 (1.08%)	0 (0.00%)	9 (1.32%)	0.1561
Have you had issues renewing your treatment at the pharmacy?	767	48 (6.26%)	3 (2.10%)	45 (7.21%)	**0.0228**
*Feelings and psychological impact about the COVID period*					
How would you rate your anxiety at present? VAS/10 scale (mean, STD)	1005	3.19 (2.65)	3.19(2.65)	3.24 (2.56)	0.5624
Patients with a"moderate"to"very high impact"(> 3) on anxiety	1005	431 (42.89%)	107 (47.14%)	324 (41.65%)	0.1413
How health crisis has affected your management? VAS/10 scale (mean, STD)	1008	1.81 (1.49)	2.21 (2.36)	1.69 (2.51)	** <.0001**
Patients with a"moderate"to"very high impact"(> 3) on amyloidosis management	1008	213 (21.13%)	60 (26.20%)	153 (19.64%)	**0.0325**
Did your amyloidosis worsen during the imposed confinement?	226	15 (6.64%)	0 (0.00%)	15 (7.61%)	0.1241
*Infection by SARS-CoV-2*					
Have you been infected with the COVID-19 virus?	972	94 (9.67%)	16 (7.05%)	78 (10.47%)	0.1268
Have you tested positive for the COVID-19 virus?	102	93 (91.18%)	16 (100.00%)	69 (88.46%)	0.1530
Have you been hospitalized as a result of COVID-19 infection?	86	38 (44.19%)	12 (75.00%)	26 (37.14%)	**0.0059**
Have you been in intensive care because of COVID-19?	42	14 (33.33%)	5 (41.67%)	9 (30.00%)	0.4687
How many nights did you stay in hospital? (mean, STD)	60	11.82 (11.30)	19.23 (10.33)	9.77 (10.78)	**0.0083**
Have you received the anti-COVID vaccine?	587	540 (91.99%)	97 (96.04%)	443 (91.15%)	0.0996
*Containment period*					
Negative impact					
On your mood	980	398 (40.61%)	107 (48.20%)	291 (38.39%)	**0.0089**
On your stress	961	329 (34.24%)	93 (43.46%)	236 (31.59%)	**0.0013**
On your psychological state	947	333 (35.16%)	97 (45.12%)	236 (32.24%)	**0.0005**
On your physical condition	958	280 (23.23%)	72 (33.64%)	208 (27.96%)	0.1069
On your amyloidosis management	947	108 (11.40%)	26 (11.98%)	82 (11.23%)	0.7606
On your sleeep	967	231 (23.89%)	59 (27.44%)	172 (22.87%)	0.1658
On your diet	958	124 (12.94%)	29 (13.55%)	95 (12.77%)	0.7638
On your amyloidosis	968	110 (11.36%)	19 (8.80%)	91 (12.10%)	0.1774
Rarely or neve respected					
Barrier gestures	1013	6 (0.59%)	1 (0.44%)	5 (0.64%)	0.7272
Hand washing	1008	8 (0.79%)	0.00%)	8 (1.03%)	0.1247
Wearing a mask	1013	3 (0.30%)	0 (0.00%)	3 (0.38%)	0.3485
Home containment	1008	15 (1.49%)	3 (1.32%)	12 (1.54%)	0.8070
Bad experience					
Home containment	993	319 (32.12%)	85 (37.95%)	234 (30.43%)	**0.0340**
Social isolation	892	320 (35.87%)	80 (41.03%)	240 (34.43%)	0.0898
Family separation	878	475 (54.10%)	127 (64.47%)	348 (51.10%)	**0.0009**
Inability to see your children	998	343 (34.27%)	73 (32.02%)	269 (34.94%)	0.4149
Inability to see your grandchildren	1001	423 (42.26%)	97 (42.73%)	326 (42.12%)	0.8695
Christmas celebration	1006	337 (33.50%)	74 (32.46%)	263 (33.80%)	0.7044

Data are presented as n (%) or mean ± SD. AL-CA indicates AL cardiac amyloidosis; ATTR-CA, Transthyretin cardiac amyloidosis

### Impact on diagnosis

**Fig. 3:**
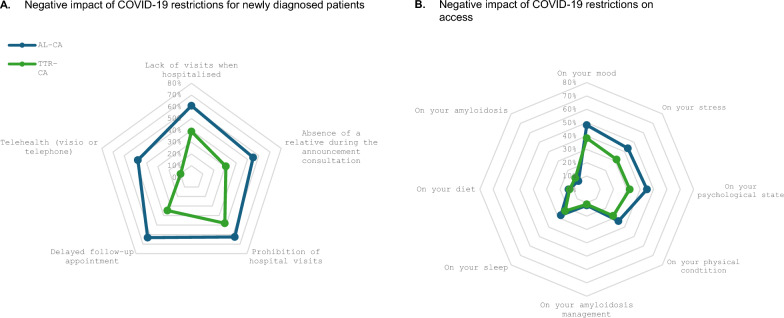
Negative impact of COVID-19 restrictions. Figure 3 illustrates the overall impact of the containment restrictions on healthcare access and quality of life. The Spider graphs representing (A) Negative impact of COVID-19 restrictions for newly diagnosed patients (B) Negative impact of COVID-19 restrictions on healthcare access. The percentages correspond to the proportion of patients with a bad or very-bad feeling for each proposition. AL-CA: AL Cardiac Amyloidosis; ATTR-CA: ATTR Cardiac Amyloidosis

222 patients (21.8% of the survey respondents) had been newly diagnosed during the lockdown period lockdown period and subsequent containment periods. Among them, 52 were AL-CA and 170 ATTR-CA. Figure 3A illustrates the impact the lockdown period had on patient feelings about their diagnosis. Overall, AL-CA patients reported having a significantly worse experience than patients with ATTR-CA. Specifically, this negative diagnostic experience was due to restrictions for hospital visitors, which was reported significantly more often among AL-CA patients than ATTR-CA patients (*p* = 0.0185). These restrictions prevented patients from having a caregiver or relative present at the time of diagnosis, which was reported significantly more often among patients with AL-CA than ATTR-CA (*p* = 0.0109). Lastly, respondents reported longer waiting times for consultation and teleconsultations worsened consulting conditions affecting AL-CA significantly more often than ATTR-CA (*p* = 0.0016, Table 1).

### Impact on patient follow-up

Overall, patient follow-up consultations were disrupted similarly between amyloidosis types, irrespective of the place of care (community, hospital or referral centre) or region (Paris metropolitan area or regional France). Fewer than one out of five patients were affected. Also, in almost one of two cases, no other alternative consultation was proposed when consultations or care were postponed (Table 1). Similarly, we found that follow-up examinations and hospitalizations were cancelled or postponed with no difference according to region of living (Supplementary Table 3).

### Impact on patient treatment

During the survey period, most patients (83.3%) were receiving either an oral or injectable treatment. However, the lockdown period did not seem to have an adverse effect on treatment. Only 6.3% of the total population reported issues renewing their medications and these issues were reported significantly more often among patients with ATTR amyloidosis (7.2%, *p* = 0.0228). Very few patients discontinued their treatment 1.1% (9/834) or reported having experienced worsening of their disease (6.6%). This was similar between amyloidosis subtypes.

### Anti-SARS-CoV-2 vaccination

Respondents were also asked whether they had received a SARS-CoV-2 vaccination. Interestingly, only 587 (57.8%) of respondents answered this question, yet almost all had been vaccinated, with no difference between Paris metropolitan area or regional France. Curiously, we observed significantly fewer patients with ATTRv had been vaccinated than patients with ATTRwt (*p* = 0.0183) (Supplementary Table 2).

### Preventive health measures to prevent SARS-CoV-2 in France and depending on area of life

Concerning preventative measures to prevent SARS-CoV-2 infection, compliance with barrier measures was excellent among those patients surveyed. Only a small minority of the population rarely or never performed barrier gestures, rarely or never hand-washed, rarely or never wore masks, and rarely or never obeyed lockdown rules. No difference was observed between subtypes.

Although few respondents reported being infected with SARS-CoV-2 virus, significantly more infections occurred among those patients living in the Paris Metropolitan Area than regional France (Supplementary Table 3). Among those who reported having been infected with SARS-CoV-2, a large proportion reported having been hospitalized 38/86 (44.19%). Of these, significantly more AL-CA patients were hospitalized than ATTR-CA; (*p* = 0.0059), (Table 1), irrespective of region (*p* = 0.0778, (Supplementary Table 3). The average declared length of hospital stay was 11.8 nights, of which, significantly longer stays were observed among patients with AL-CA and in the Paris Metropolitan Area (Supplementary Table 3). Among those patients who were hospitalized for SARS-CoV-2 infection, around a third of them were admitted to intensive care, with no difference by amyloidosis subtype, or region. (Supplementary Table 2 and 3).

### Psychological impact of lockdown

The level of anxiety respondents reported was moderate to high, > 3 on a visual analogue scale for 431/1005 (42.9%) of the population, although there was no difference between CA subtypes (Table 1).

Figure 3B illustrates the main negative dimensions patients reported experiencing during the lockdown and containment period. Overall, around a third of respondents reported their experience of lockdown as being negative or very negative, especially among AL-CA patients (Table 1) and patients in regional France (Supplementary Table 3). These experiences differed significantly between AL-CA and ATTR-CA patients and according to the region in which they live. Specifically, significantly more AL-CA patients, reported COVID19 had an effect on their morale, psychological state and stress (Table 1).

However, sleep, physical condition, diet, and overall amyloidosis management was less impacted, and no difference was observed between ATTR-CA and AL-CA patients. However, more specifically, we observed that ATTRv were significantly more affected in mood, stress and psychological state than ATTRwt (Supplementary Table 2).

## Discussion

This survey explored the impact of the reduced healthcare access due to the COVID-19 lockdown and containment periods in a large cohort of cardiac amyloidosis patients. We estimate the questionnaire was answered by approximately one-sixth of the total French population of cardiac amyloidosis at that time. It showed that cardiac amyloidosis patients felt the management of their disease had been negatively affected during this health crisis irrespective of amyloidosis type. This is the first report, to our knowledge, to describe the impact of the constraints associated with COVID-19 from the perspective of a CA patient. Specifically, around a fifth of patients surveyed reported feeling that the health crisis caused by the COVID-19 pandemic significantly disrupted their healthcare, of which significantly more were AL-CA patients. Yet in practice, these patients were generally prioritised considering the seriousness of their disease. They were allowed to attend hospital visits and chemotherapy sessions. Possibly, this negative view may be due to their awareness of the severity of their disease.

Similarly, newly diagnosed AL-CA patients reported having a poor inpatient experience during their inpatient stay for their initial work-up. This negative experience was a direct consequence of containment measures which prevented patients from receiving hospital visits at this time. Also, this experience may have been exacerbated by diagnosis delays probably due to the relative rarity of this condition, heterogeneous clinical picture and the difficulty to program multiple organ function tests at this time [13]. However, we were unable to measure the impact on patients who were not diagnosed during this period and who may have subsequently died.

Concerning current CA patients, the COVID-19 lockdown minimally impacted clinical follow-up irrespective of region or healthcare centre. Although a small proportion of CA patients had their follow-up visit postponed, independent of amyloidosis type, region or hospital and nearly a half of them were not offered an alternative consultation, yet very few discontinued their CA treatment and reported a worsening of their disease. This finding is interesting and may be useful to consider improving efficiency of care moving forward post-COVID-19, considering the role telemedicine is taking in managing patients with heart failure [14, 15]. Although few cardiologists used teleconsultation for patient follow up before the COVID-19 pandemic, once it was established patients remained stable, they gained a level of responsibility and autonomy for their disease. This suggests that going forward, telemedicine may be an alternative way to monitor certain patients. Indeed, teleconsultation for patients with heart failure offers numerous benefits for both the patient and the practitioner, foremost among them being the maintenance of the relationship between healthcare providers and patients. Furthermore, during a pandemic, it allows for a reallocation of resources, which is particularly relevant in times of restricted access to hospitals and increased patient flow, as we have experienced [16]. Lastly, we can also anticipate that in the future, digital biomarkers (for example, collected via smartphones) could enable functional assessment of patients in their daily lives. Applied to amyloidosis, we can hypothesize that these new tools could help in monitoring disease progression as the Six-Min Walk Test does today, for instance [17].

Importantly, around a third of respondents reported their experience during lockdown as being negative, which is similar to reports of patients living with heart failure of other aetiology [18]. Specifically, AL-CA and those patients living in Paris metropolitan area reported lower morale, psychological state and increased stress. Although these feelings were also reported in the general community [19], they were particularly acute among AL-CA patients who were facing a rare and serious cardiac disease without the usual amount of support or information. Notably, the level of anxiety respondents reported was moderate to high, for a large proportion of the population, which was similar between CA subtypes. This concords with the literature, in which patients describe the impact of cardiac amyloidosis as being associated with high levels of anxiety. One recent survey reported that 58% of patients with CA reported anxiety or depression [9], similar to findings showing 37% of newly diagnosed AL amyloidosis patients had depression and 47% had anxiety measured using the SF-36 depression scale [20]. This is important because there is a relationship between symptom severity and psychological disorders, which can be mediated by life satisfaction. This highlights the need for systematic, psychological support for these patients, particularly at diagnosis considering cardiac symptom onset and symptom severity. This support could be provided within the care centre and continuing support could be provided by local patient networks within a national patient association.

Concerning SARS-COV-2 infection, very few patients reported having been infected. However, among those who were infected, significantly more AL-CA patients were admitted and remained in hospital longer than those with ATTR-CA. The hospital stay length was significantly longer in the Paris metropolitan area than regional France. This was expected considering the additional risk this population was of being infected due to their immunocompromised state and is similar to other retrospective reports [21, 22]. Also, the number of patients having reported a SARS-COV-2 infection was noticeably higher among those living in the Paris metropolitan region than those in small regional areas [23]. This highlights the importance of paying particular attention to infectious diseases in the AL-CA population, particularly those living in densely populated metropolitan areas.

Interestingly, among the 587 patients (57.0%) who answered the question about vaccination, 92.0% reported to have been vaccinated which is in line with the general population vaccination (91.8%) among people aged between 70 and 74 years [24]. It is not known whether those patients who declined to respond had been vaccinated. This was rather surprising considering that, vaccination was supported for this vulnerable patient population but may correspond to a population of patients with vaccine hesitancy [21].

There are several limits to this survey, related to the subjective nature of a respondent completed questionnaire, which may have induced recall bias. As the questionnaire was disseminated after the last lockdown it is possible that patient memory of events was inaccurate. However, considering the extraordinary circumstances and the short delay between the end of the lockdown period and study start, it is unlikely many errors were induced. Also, despite the concerted effort and cooperation between the national expert centres, networks and patient association to diffuse the questionnaire to approximately a quarter of the CA population in France, many CA patients may not have been aware of the survey. Also, this patient perspective was limited to those patients who survived the first lockdown when no masks or vaccines were available. Nevertheless, for a rare disease this is a very strong sample size which is similar to other surveys performed among CA or heart failure patients at this time [25].

## Conclusion

This study shows that the COVID-19 pandemic had a negative effect on mental well-being among all CA patients. Although clinical follow-up was disrupted, treatment was maintained. This suggests that telemedicine could be maintained for some stable patient groups. However, newly diagnosed AL-CA patients were particularly affected due to the lack of support in hospital.

## Supplementary Information

Additional file 1.

Additional file 2. 

Additional file 3.

## Data Availability

The dataset supporting the conclusions of this article is included within the article and supplementary tables.

## Abbreviations

AL: Light chain amyloidosis
AL-CA: Light chain cardiac amyloidosis
ATTR-CA: Transthyretin cardiac amyloidosis
ATTR: Transthyretin amyloidosis
ATTRv: Hereditary Transthyretin amyloidosis
ATTRwt: Wild-Type Transthyretin amyloidosis
CA: Cardiac amyloidosis (CA)
TTR: Transthyretin
