# Supplementary material for: The impact of limited healthcare access among patients with light chain and transthyretin amyloidosis: real-world survey during COVID-19 lockdown period in France
Source: Orphanet J Rare Dis. 2025 Jul 8;20:347. doi: 10.1186/s13023-025-03859-1 (PMC12235858; doi:10.1186/s13023-025-03859-1)
Supplement: Supplementary file 1 — Additional file 1. [file 13023_2025_3859_MOESM1_ESM.docx]

| Supplementary Table 1. Self- reported questionnaire | | | | | | | | | | | | | | | | | | | | | | | |
| --- | --- | --- | --- | --- | --- | --- | --- | --- | --- | --- | --- | --- | --- | --- | --- | --- | --- | --- | --- | --- | --- | --- | --- |
| **Thank you very much for completing this questionnaire.**  **You can send it back by mail to your cardiologist** | | | | | | | | | | | | | | | | | | | | | | | |
| **This questionnaire is anonymous and short. It fills up in 3min.**  **It was carried out by the Amyloidosis Network and has the support of the French Association Against Amyloidosis, the Rare Diseases Network CARDIOGEN, the Amyloidosis Network, the CNCF and the GICC.**  **Please take the time to fill it out and send it back to your cardiologist** | | | | | | | | | | | | | | | | | | | | | | | |
| **1** | **Which French region do you live in? *(Check only one box)*** | | | | | | | | | | | | | | | | | | | | | | |
|  | Auvergne-Rhône-Alpes  Bourgogne-Franche-Comté  Brittany  Centre-Val de Loire | | Corsica  Grand Est  Hauts-de-France  Île-de-France | | | Normandy  Nouvelle-Aquitaine  Occitanie  Pays de la Loire | | | | | | | | Provence-Alpes-Côte d'Azur  DOM -TOM | | | | | | | | | |
| **2** | **What is your gender? *(Check only one box)*** | | | | | | | | | | | | | | | | | | | | | | |
|  | Male; Female | | | | | | | | | | | | | | | | | | | | | | |
| **3** | **Could you specify your age (in years)?** | | | | | | | | | | | | | | | | | | | | | | |
|  | ___years | | | | | | | | | | | | | | | | | | | | | | |
| **4** | **Could you elaborate, what form of amyloidosis do you have? *(Check only one box)*** | | | | | | | | | | | | | | | | | | | | | | |
|  | Senile wild-type transthyretin amyloidosis.  Hereditary (genetic) transthyretin amyloidosis.  Hematologic AL amyloidosis (treated with chemotherapy).  AA amyloidosis | | | | | | | | | TTR cardiac amyloidosis with ongoing genetic analysis.  I don't know what my type of amyloidosis is  I'm not sure if I have amyloidosis | | | | | | | | | | | | | |
| **5** | **Was your amyloidosis diagnosed during the COVID19 health crisis? *(Check only one box)*** | | | | | | | | | | | | | | | | | | | | | | |
|  | Yes; No | | | | | | | | | | | | | | | | | | | | | | |
| ***If yes, answer question 5, answer question 6, if "no", go to question 7*** | | | | | | | | | | | | | | | | | | | | | | | |
| **6** | **If you were diagnosed with amyloidosis during the attack, how did you experience the following?** | | | | | | | | | | | | | | | | | | | | | | |
|  | ***Check only one box for each proposal*** | | | | | | | | | | Very badly experienced | Poorly experienced | | | | Neither good nor badly lived | | | Well lived | | | | Not applicable |
|  | Absence of visits in case of hospitalization | | | | | | | | | |  |  | | | |  | | |  | | | |  |
|  | The absence of the spouse or family at the time of the advertisement consultation | | | | | | | | | |  |  | | | |  | | |  | | | |  |
|  | Ban on hospital visits | | | | | | | | | |  |  | | | |  | | |  | | | |  |
|  | Longer wait times to get an appointment. | | | | | | | | | |  |  | | | |  | | |  | | | |  |
|  | Consultations by video or phone | | | | | | | | | |  |  | | | |  | | |  | | | |  |
| **7** | **Because of the COVID pandemic, have your medical consultations at the CITY OFFICE been postponed? *(Check only one box)*** | | | | | | | | | | | | | | | | | | | | | | |
|  | No, I went Yes  , I cancelled or rescheduled my appointment | | | | | | Yes, my doctor cancelled or postponed it  No, I didn't have an appointment. | | | | | | | | | | | | | | | | |
| **8** | **If your consultation at the CITY OFFICE has been cancelled or postponed, has your doctor suggested that you  *(several possible choices, so you can check several boxes):*** | | | | | | | | | | | | | | | | | | | | | | |
|  | A teleconsultation (with Visio)  A telephone consultation | | | | | | My  doctor didn't offer me anything | | | | | | | | | | | | | | | | |
| **9** | **Because of the COVID pandemic, have your consultations in an expert center been postponed? *(Check only one box)*** | | | | | | | | | | | | | | | | | | | | | | |
|  | No, I went  Yes, I cancelled or rescheduled my appointment | | | | | | Yes, my doctor cancelled or postponed it  No, I didn't have an appointment. | | | | | | | | | | | | | | | | |
| **10** | **If your EXPERT CENTER CONSULTATION has been cancelled or postponed, has your EXPERT CENTER offered you *(Several choices possible, so you can tick several boxes)*** | | | | | | | | | | | | | | | | | | | | | | |
|  | A teleconsultation (with Visio)  A telephone consultation | | | | | | My  doctor didn't offer me anything | | | | | | | | | | | | | | | | |
| **11** | **Because of the COVID pandemic, have your visits to the hospital for the follow-up of your amyloidosis been cancelled or postponed? *(Check only one box)*** | | | | | | | | | | | | | | | | | | | | | | |
|  | No, I went Yes  , I cancelled or rescheduled my appointment | | | | | | Yes, my doctor cancelled or postponed it  No, I didn't have an appointment. | | | | | | | | | | | | | | | | |
| **12** | **If your consultation has been cancelled or postponed, has THE HOSPITAL offered you *(Multiple choices possible, so you can tick several boxes)*** | | | | | | | | | | | | | | | | | | | | | | |
|  | A teleconsultation (with video)  A telephone consultation | | | | | | An email consultation  The hospital didn't offer me anything | | | | | | | | | | | | | | | | |
| **13** | **Due to the COVID pandemic, have your amyloidosis EXAMS been cancelled or postponed? *(Check only one box)*** | | | | | | | | | | | | | | | | | | | | | | |
|  | No, my exams took place Yes  , I cancelled or postponed my exams | | | | | | Yes, my exams were cancelled or postponed  No, I didn't have an appointment. | | | | | | | | | | | | | | | | |
| **14** | **Because of the COVID pandemic, have your HOSPITALIZATIONS for the follow-up of your amyloidosis been cancelled or postponed? *(Check only one box)*** | | | | | | | | | | | | | | | | | | | | | | |
|  | No, I went  Yes, I cancelled or postponed my hospitalization | | | | | | Yes, my doctor cancelled or postponed it  No, I didn't have a planned hospitalization | | | | | | | | | | | | | | | | |
| **15** | **Do you (during the COVID19 pandemic) have oral or injectable treatment for your amyloidosis? *(Check only one box)*** | | | | | | | | | | | | | | | | | | | | | | |
|  | Yes an oral treatment  Yes an injectable treatment | | | | | | Yes an oral and injectable treatment  I have no treatment | | | | | | | | | | | | | | | | |
| **16** | **Did you (during the COVID19 pandemic) stop your treatment for amyloidosis?*(Check only one box)*** | | | | | | | | | | | | | | | | | | | | | | |
|  | Yes because I was worried about the dangers of the treatment  Yes on the advice of my doctor  Yes because the treatment is no longer available at the pharmacy | | | | | | No, I continued my treatment in the same way  Yes because I was not able to go to the pharmacy  Not concerned | | | | | | | | | | | | | | | | |
| **17** | **Have you had trouble renewing your treatment at the pharmacy? *(Check only one box)*** | | | | | | | | | | | | | | | | | | | | | | |
|  | Yes; No; Not applicable | | | | | | | | | | | | | | | | | | | | | | |
| **18** | **Currently, how would you situate your anxiety?**  ***Please draw a vertical line between 0 and 10 to answer.*** | | | | | | | | | | | | | | | | | | | | | | |
|  | No  anxiety | 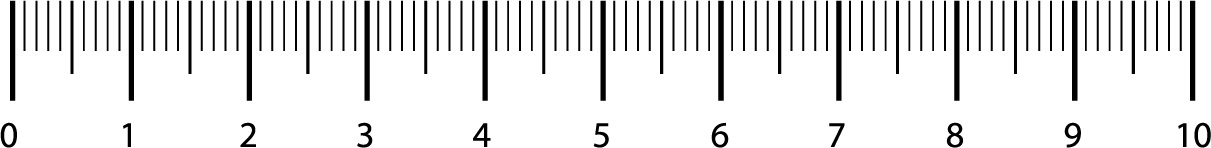 | | | | | | | | | | | | | | | | | | Maximum anxiety | | | |
| **19** | **In your opinion, has the health crisis disrupted the management of your amyloidosis?**  ***Please draw a vertical line between 0 and 10 to answer.*** | | | | | | | | | | | | | | | | | | | | | | |
|  | No  disruption | 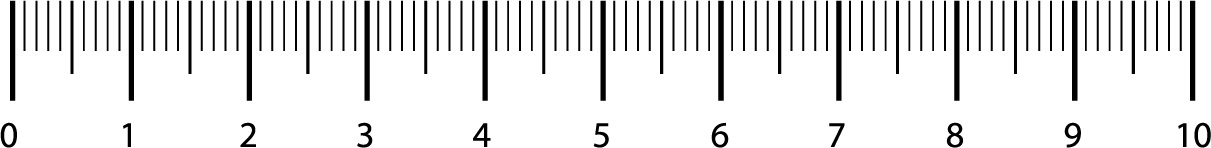 | | | | | | | | | | | | | | | | | | Maximum Disruption | | | |
| **20** | **Has your amyloidosis worsened during the lockdown imposed by health authorities? *(Check only one box)*** | | | | | | | | | | | | | | | | | | | | | | |
|  | Yes; No; I can't say | | | | | | | | | | | | | | | | | | | | | | |
| **21** | **Yourself, Have you been infected with the COVID-19 virus? *(Check only one box)*** | | | | | | | | | | | | | | | | | | | | | | |
|  | Yes; No; I can't say | | | | | | | | | | | | | | | | | | | | | | |
| **22** | **Yourself, Have you tested positive for the COVID-19 virus? *(Check only one box)*** | | | | | | | | | | | | | | | | | | | | | | |
|  | Yes; No | | | | | | | | | | | | | | | | | | | | | | |
| **23** | **Have you been hospitalized due to Covid 19? *(Check only one box)*** | | | | | | | | | | | | | | | | | | | | | | |
|  | Yes; No | | | | | | | | | | | | | | | | | | | | | | |
| **24** | **Have you been in intensive care due to Covid 19? *(Check only one box)*** | | | | | | | | | | | | | | | | | | | | | | |
|  | Yes; No | | | | | | | | | | | | | | | | | | | | | | |
| **25** | **If hospitalized, How many nights were you in the hospital? *(Indicate number of nights)*** | | | | | | | | | | | | | | | | | | | | | | |
|  | ___Nights | | | | | | | | | | | | | | | | | | | | | | |
| **26** | **Has a loved one been infected with the COVID-19 virus?**  ***By relative, we mean a person who lives in the same place as you or a person you see more than 3 times a week.*** | | | | | | | | | | | | | | | | | | | | | | |
|  | Yes; No | | | | | | | | | | | | | | | | | | | | | | |
| **27** | **Has a loved one tested positive for the COVID-19 virus? *(Check only one box)*** | | | | | | | | | | | | | | | | | | | | | | |
|  | Yes; No | | | | | | | | | | | | | | | | | | | | | | |
| *If you checked "no" in question 27, go directly to question 31If you checked "yes" in question 27, don't forget to answer questions 28 to 30.* | | | | | | | | | | | | | | | | | | | | | | | |
| **28** | **If yes to question 27, has this relative been hospitalized due to the COVID19? *(Check only one box)*** | | | | | | | | | | | | | | | | | | | | | | |
|  | Yes; No | | | | | | | | | | | | | | | | | | | | | | |
| **29** | **If yes to question 27, has this relative been in the intensive care unit? *(Check only one box)*** | | | | | | | | | | | | | | | | | | | | | | |
|  | Yes; No | | | | | | | | | | | | | | | | | | | | | | |
| **30** | **If yes to question 27, how many nights did your loved one stay in the hospital because of the COVID19? *(Check only one box)*** | | | | | | | | | | | | | | | | | | | | | | |
|  | Yes; No | | | | | | | | | | | | | | | | | | | | | | |
| **31** | **Have you received the COVID19 vaccine *(Check only one box)*** | | | | | | | | | | | | | | | | | | | | | | |
|  | Yes; No | | | | | | | | | | | | | | | | | | | | | | |
| **32** | **How many doses of COVID19 vaccine have you received? *(Check only one box)*** | | | | | | | | | | | | | | | | | | | | | | |
|  | One | | | Two | | | | | | | | | Three or more | | | | | | | | | | |
| **33** | **What is the approximate date of your first dose? *(Indicate day and month)*** | | | | | | | | | | | | | | | | | | | | | | |
|  | _ _ / _ _ / 2021 (day/month/year) | | | | | | | | | | | | | | | | | | | | | | |
| **34** | **In your opinion, have all these periods of confinement had an impact?** | | | | | | | | | | | | | | | | | | | | | | |
|  | *Check only one box for each proposal* | | | | | | | Yes, a negative impact | | | | Yes, a positive impact | | | | | No impact | | | | | I can't say | |
|  | On your morale | | | | | | |  | | | |  | | | | |  | | | | |  | |
|  | On your stress | | | | | | |  | | | |  | | | | |  | | | | |  | |
|  | On your psychological state | | | | | | |  | | | |  | | | | |  | | | | |  | |
|  | On your physical condition | | | | | | |  | | | |  | | | | |  | | | | |  | |
|  | On the management of your amyloidosis | | | | | | |  | | | |  | | | | |  | | | | |  | |
|  | On Your Sleep | | | | | | |  | | | |  | | | | |  | | | | |  | |
|  | On your diet | | | | | | |  | | | |  | | | | |  | | | | |  | |
|  | Overall on your amyloidosis | | | | | | |  | | | |  | | | | |  | | | | |  | |
| **35** | **In recent months, have you complied with the following measures:** | | | | | | | | | | | | | | | | | | | | | | |
|  | *Check only one box for each proposal* | | | | Systematically | | | | | | As often as possible | | | | When necessary | | | | | | Rarely or Never | | |
|  | Barrier gestures | | | |  | | | | | |  | | | |  | | | | | |  | | |
|  | Hand washing | | | |  | | | | | |  | | | |  | | | | | |  | | |
|  | Wearing a mask | | | |  | | | | | |  | | | |  | | | | | |  | | |
|  | Lockdown | | | |  | | | | | |  | | | |  | | | | | |  | | |
| **36** | **Do you have loved ones who have died from COVID? *(Check only one box)*** | | | | | | | | | | | | | | | | | | | | | | |
|  | Yes; No | | | | | | | | | | | | | | | | | | | | | | |
| **37** | **Can you tell us what your ties are with your loved ones (friends, brother/sister, cousin, parents...)?** | | | | | | | | | | | | | | | | | | | | | | |
|  | **__** | | | | | | | | | | | | | | | | | | | | | | |
| **38** | **All in all, how did you experience the following situations?** | | | | | | | | | | | | | | | | | | | | | | |
|  | *Check only one box for each proposal* | | | | Very badly experienced | | | | Poorly experienced | | | Neither good nor badly lived | | | | | | Well lived | | | | Not applicable | |
|  | Containment | | | |  | | | |  | | |  | | | | | |  | | | |  | |
|  | Isolation | | | |  | | | |  | | |  | | | | | |  | | | |  | |
|  | Separation from family | | | |  | | | |  | | |  | | | | | |  | | | |  | |
|  | Inability to see children | | | |  | | | |  | | |  | | | | | |  | | | |  | |
|  | Inability to see grandchildren | | | |  | | | |  | | |  | | | | | |  | | | |  | |
|  | Christmas celebrations | | | |  | | | |  | | |  | | | | | |  | | | |  | |
