# Supplementary material for: The impact of limited healthcare access among patients with light chain and transthyretin amyloidosis: real-world survey during COVID-19 lockdown period in France
Source: Orphanet J Rare Dis. 2025 Jul 8;20:347. doi: 10.1186/s13023-025-03859-1 (PMC12235858; doi:10.1186/s13023-025-03859-1)
Supplement: Supplementary file 3 — Additional file 3. [file 13023_2025_3859_MOESM3_ESM.docx]

**Supplementary Table 3 -** Impact of the COVID-19 pandemic on patients according to urban or regional residency.
